# The Effect of Hypo-Hydration on Mood and Cognition Is Influenced by Electrolyte in a Drink and Its Colour: A Randomised Trial

**DOI:** 10.3390/nu11092002

**Published:** 2019-08-24

**Authors:** Alecia L. Cousins, Hayley A. Young, Andrew G. Thomas, David Benton

**Affiliations:** Department of Psychology, Swansea University, Swansea SA2 8PP, UK

**Keywords:** anxiety, cognition, colour of drink, dehydration, electrolyte, fluid intake, mood, placebo, rehydration

## Abstract

Traditionally, it has been thought necessary to lose 2% of body mass due to dehydration to disrupt functioning, although recently, adverse effects have been reported, with a loss of 0.5%–0.7%. It is, however, unclear whether the response to small reductions in mass reflects dehydration as homeostatic mechanisms are thought to be effective. As psychological responses are most commonly reported, it is strange that the possibility of a placebo response has not been considered. Individuals were therefore subject to a temperature of 30 °C for three hours, and mood and cognition were monitored. To consider changes in hydration status, drinks were compared, differing in their ability to rehydrate due to the presence or absence of electrolytes. The possibility of a placebo response was considered by comparing the response to plain or coloured water. Not drinking was disruptive, although a combination of plain water and electrolyte tended to be the most effective means of preventing a decline in mood, indicating a role for rehydration after a loss of 0.66% body mass. There was, however, also evidence of a placebo response: a combination of plain water and electrolyte tended to be better able to prevent a decline in mood than coloured water and electrolyte.

## 1. Introduction

It has been suggested that a loss of body mass of over 2% is necessary to disrupt athletic performance [1], mood [2], attention, executive functioning and motor coordination [3]. However, more recently, a series of studies have found a disruption of psychological functioning [4] and driving [5], with a loss of body mass of 1% or less. In addition, in intervention studies, the cognition of children improved after consuming water [6,7,8,9].

The first aim was to explore the mechanisms involved when changes in psychological functioning result from a small reduction in body mass. Although homeostatic mechanisms are known to be efficient and hypo-hydration has been defined as a loss of more than 2% body mass, a loss of about 0.6% was considered in the present study. Where the term hypo-hydration is used presently, it implies a change of this smaller magnitude. By examining drinks that were predicted to differentially rehydrate, that is, they did or not contain electrolyte, the hypothesis was considered that dehydration as such played a role in the consequences associated with minor changes in hydration status.

In addition, as the initial changes associated with hypo-hydration have been reported to be most often psychological, it is relevant that Liberman [10] noted that factors not usually considered when examining physiology come into consideration. The behaviour of humans is influenced by their own expectations and the suggestions and cues of others. However, the possibility that a placebo response to a drink was responsible for psychological changes has not to date been examined. One objective was therefore to consider the possibility that psychological responses to drinking when hypo-hydrated, in part at least, reflected expectation.

If psychological mechanisms were associated with hypo-hydration, then events that had been associated historically with rehydration may be influential. For example, the sight of drinks that had previously resulted in rehydration could stimulate anticipatory changes. One possible stimulus could be the colour of a liquid; in particular, sports, energy and fruit drinks have a characteristic appearance. There is considerable evidence that the response to food has a cephalic phase in that the brain responds to the smell and sight of food by initiating physiological responses that anticipate consumption [11]. These changes, amongst others, include alterations in the secretion of hydrochloric acid, gastrin, lipase, gherlin and insulin. Compared with food, there have been fewer studies of drink, although there is no reason to suggest that the appearance of drink might not play a role. Therefore, the colour of a drink was considered, as the colour of a placebo is particularly influential [12]. For the first time, changes in mood and cognition were found associated with the colour of a drink, although in addition, physiological mechanisms were influential.

Thus, the aim was firstly to consider whether rehydration played a role when there are small changes in body mass by comparing drinks differing in their ability to rehydrate. Secondly, the possibility of a placebo effect was examined by changing the appearance of a drink.

## 2. Materials and Methods 

### 2.1. Subjects

Participants were recruited using email and posters in Swansea University. Using G*Power [13], the sample size was calculated to consider both within- and between-subject effects. The parameters were six groups with an expected correlation of 0.6, a probability of <0.05, a two tailed test and 80% power to detect medium to large effects (Cohen’s f = 0.4). It was estimated that a sample of 162 was required: in the event, 174 subjects were recruited. The sample had a mean age of 20.1 (SD 2.4) years, of which 85 were male and 89 females. The BMI of the males was 23.7 (4.1) and females 23.0 (4.2). Inclusion criteria were 18 to 30 years, non–smokers, BMI < 30 and being in good health by self-report and not taking medication for any psychological or other health disorder. Approval was obtained from the research ethics committee of the Department of Psychology, Swansea University: subjects gave written informed consent and were paid £40 for participating. The trial was registered with Clinicaltrials.gov, NCT03948230.

### 2.2. Design

The experimental conditions were created from combinations of two types of capsules (containing 300 mg of sodium chloride or corn flower as a placebo) and three types of drink (No drink, 300 mL plain water; 300 mL of plain water to which was added purple/dark red food colourant (Dr. Oetker, Bielefeld, Germany)). There resulted six experimental conditions: (1) Electrolyte capsule plus no water; (2) placebo capsule plus no water; (3) electrolyte capsule plus 300 mL of plain water; (4) placebo capsule plus 300 mL of plain water; (5) 300 mL coloured water plus electrolyte capsule; (6) 300 mL coloured water plus placebo capsule. Those only receiving a capsule swallowed them without liquid. The sequence in which the various conditions were allocated was determined using a computer based random number generator and in a parallel design, they were given in the order that subjects were recruited. DB was responsible for randomization and AC recruited the subjects. Both subjects and researchers were blind to the content of the capsules and the subjects were unaware that others were or were not consuming a drink and that the nature of the drinks differed.

### 2.3. Procedure

Participants arrived at the laboratory wearing light clothing. For the duration of the study, the room temperature was 30 degrees centigrade. To provide findings representative of everyday life, they were instructed to consume their normal breakfast before arrival. Between 09:00 and 09:30, a baseline test battery was completed and at 09:45, the first drink/capsules were consumed. At 10:30, the test battery was completed a second time, followed at 11:15 with the consumption of the second drink/capsule. The test battery was taken on a final occasion beginning at 12:00.

### 2.4. Osmolality

A urine sample was provided on arrival and again at the end of the study session. Samples were analysed using an Osmat3000 (GoNotec GmbH, Berlin, Germany), which uses a freezing point osmometer to determine milliOsmoles/kilogram.

### 2.5. Body Mass

Body mass was measured with the use of an electronic scale (Kern KMS-TM; Kerr and Sohn GmbH, Berlin, Germany) that, to avoid problems associated with movement, took 50 assessments over a five second period and produced a mean value. The scale weighed to the nearest 5 g (17% of 1 oz) and could pick up, over short periods, changes in body mass due to breathing and perspiration. Preliminary studies examined the possible need to weigh individuals naked in case a loss of mass had been masked by perspiration remained in clothing. With the use of the current protocol, the percentage reduction in the body mass of 32 subjects who had not drunk, when weighed naked, was 0.26% (SD 0.05) after 230 min, and 0.60% (0.33) following urination. In the same individuals, these values were virtually identical when wearing light clothing; the comparable percentages of change were 0.26% (0.07) and 0.61% (0.35). Therefore, the data presented were obtained with subjects who wore the same light clothing throughout the procedure. Subjects were weighed at baseline and 180 min later having urinated on both occasions. Changes in mass from baseline to 180 min reflected fluid loss due to perspiration, breathing, and urine excretion.

### 2.6. Mood

Participants indicated the extent to which the adjectives at each end of 100-mm scales reflected their current mood. Based on the dimension of the Profile of Mood Scale [14], six lines on visual analogue scales were anchored with Agreeable–Hostile, Confused–Clearheaded, Elated–Depressed, Composed–Anxious, Unsure–Confident, Energetic–Tired. Scores were measured on a scale from 0 to 100 mm by measuring where participants placed a cross. Change scores were calculated between baseline and 90 min, and baseline and 180 min. A decrease in the scores reported indicated a decline in mood.

### 2.7. Episodic Memory: Word-List Recall

Using the MRC Psycholinguistic Database [15], three lists of 30 words were constructed, matched for the number of syllables, image-ability, and the frequency that they occur in English. With the use of a recorder, words were presented at a rate of one word every two seconds. Immediately after the presentation, participants were asked to write down as many words as they could remember (immediate recall). Approximately 20 min later, subjects were again asked to recall as many words as possible (delayed recall). The data presented are the changes in performance from baseline, where a minus score indicated poorer memory.

### 2.8. Focused Attention: Arrow Flanker Test

A modified version of the Eriksen and Eriksen [16] flanker task was used to measure the ability to focus attention and ignore peripheral information. Participants were required to indicate whether the middle arrow in a row of five was pointing to the right or left by pressing the corresponding arrow on a keyboard. On either side of the central arrow were distractors that produced a task of increasing difficulty. Congruent arrows (pointing in the same direction—<<<<<) provided the easiest version; a neutral version with squares as distractors was of intermediate difficulty (□□<□□); the most difficult version used incongruent arrows (they pointed in the opposite direction—<<><<). A stimulus remained on screen for 1.8 s or until a key was pressed. There was a randomly varying inter-stimulus interval between 1 and 3 s, with an average of 2 s. Sixty stimuli were presented pseudo-randomly, with congruent, incongruent, or neutral stimuli each appearing on 20 occasions. The reported scores were differences in average response time in milliseconds, from baseline to 90 and 180 min.

### 2.9. Working Memory

To obtain a measure of working memory, participants completed a computerised version of the serial sevens task. Participants were presented with 28 sequences of numbers, ranging between 800 and 999, with the objective of indicating whether a subsequent number was 7 less than the previous number. Changes in response times between baseline and 90 and 180 min were reported.

### 2.10. Reaction Time

Reaction times were measured using the procedure of Jensen [17]. Eight lamps were arranged in a semi-circle, equidistant (1650 mm) from a “home” key. A single trial consisted of the subject placing a finger on the “home” key: following an auditory warning signal, and after a random interval of 1 to 4 s, one of the lamps was illuminated. The light was extinguished by moving the finger from the home key to a button directly below the lamp. Anticipatory responses were impossible as if the finger left the “home” key before a lamp was illuminated, the trial was repeated. All the subjects completed a practice session of 20 trials using all eight lamps. Simple reaction times were then measured for 20 trials when only one lamp could illuminate. Choice reaction times were then measured on three occasions, when for twenty trials on each occasion 2, 4 or 8 lamps could illuminate. Decision times, that is the time to remove the finger from the home key, were reported.

### 2.11. Data Analysis

Data were analysed using a series of repeated measures analyses of variance using SPSS 22 (IBM Corporation). All data variables were screened for outliers and anomalies using Cook’s distance analysis, with the threshold calculated by dividing 4 by the number of participants (4/*N*). Initially the Mauchly’s test of sphericity was used to establish whether assumptions had been violated; where necessary, a Greenhouse-Geisser correction was applied. The Consort summary can be found in the supplementary information. Where interactions occurred, paired comparisons with Bonferroni corrections were calculated. A priori, it was decided to compare the influence of the six interventions to establish the influence of particular drinks.

## 3. Results

For brevity, only the findings associated with the type of drink or capsule are reported, although the full analyses of variance outputs are available as Supplementary Materials (Tables S1–S10). When effects concerning these two variables are not mentioned, it should be assumed that they were non-significant. Preliminary analysis found that there were no sex differences and for clarity of presentation, sex was not included in the reported results.

### 3.1. Effects of Interventions on Osmolality and Body Mass

#### Change in Hydration

To assess changes in hydration, measures of urine osmolality were obtained at baseline and the end of the study. Measures of body mass were also obtained to establish the percentage of lost body mass. The changes associated with the six interventions are reported in Table 1.

Baseline levels of osmolality did not differ between the six types of intervention. A two-way analysis of variance, Capsule (Electrolyte, placebo)×Drink (No water, water, coloured water) produced a significant effect of Drink (*F*(2, 159) = 12.84, *p* < 0.001), where those who did not receive water had a greater loss of body mass than those who received either plain or coloured water. The effect of Capsule was non–significant (*F*(1, 159) = 0.02, n.s.) (Table 1).

The mean losses of body mass for each group are presented in Table 1. A analysis of changes in osmolality over the morning again produced a significant effect of drink (*F*(2, 155) = 7.84, *p* < 0.001), where those who did not receive water had a greater increase in osmolality compared to those who drank water (plain or coloured).

### 3.2. The Influence of Six Interventions on Mood

To assess the effects of the interventions on mood, a three-way analysis of variance was conducted on each of the six mood dimensions: Drink (no water, water, coloured water) × Capsule (placebo, electrolyte)×Time (change from baseline after 90 and 180 min), with the last factor as a repeated measure.

#### 3.2.1. Agreeable

**Figure 1:**
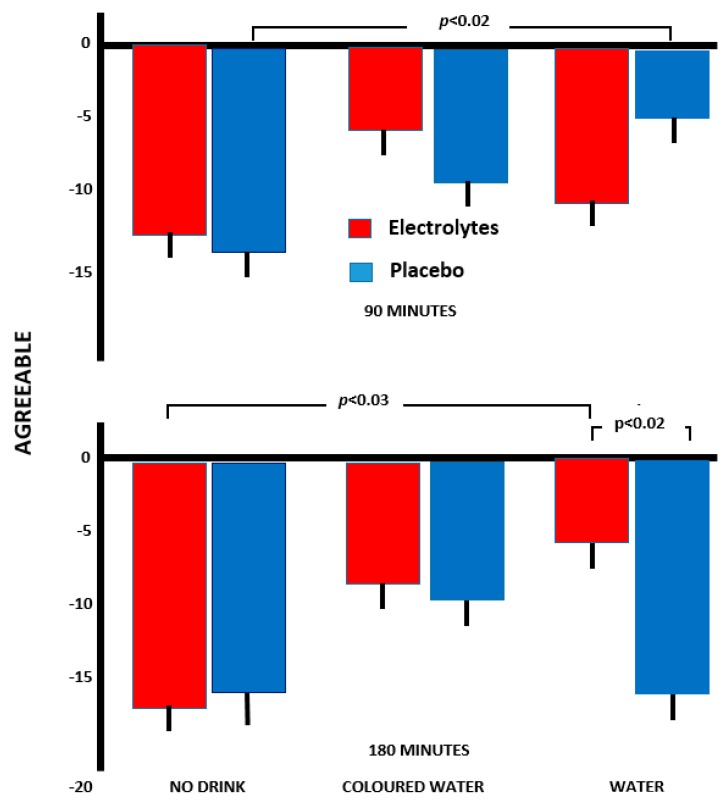
The influence of various drinks on changes over the morning in agreeableness. The data are means +/− standard errors.

A significant three way interaction Drink×Capsule×Time was found (*F*(2,154) = 4.51, *p* < 0.01) and the simple effects were analysed to explore the interaction. At 90 min, participants who received water plus placebo had the least decline in agreeability, which was significantly less than those who received a placebo capsule alone (*p* < 0.02; Figure 1). All other pairwise comparisons were non–significant.

However, after 180 min, those who received a combination of electrolyte and water had the least decline in agreeability, a decline that was significantly lower than for those who received electrolyte plus no drink (*p* < 0.03) or water plus placebo (*p* < 0.02). Thus, while electrolyte alone or water alone had no positive impact on ratings of agreeability, a combination of electrolyte plus water proved beneficial, although the same effect was not observed with coloured water and electrolyte.

A minus score indicates a decrease during the study.

#### 3.2.2. Composure

**Figure 2:**
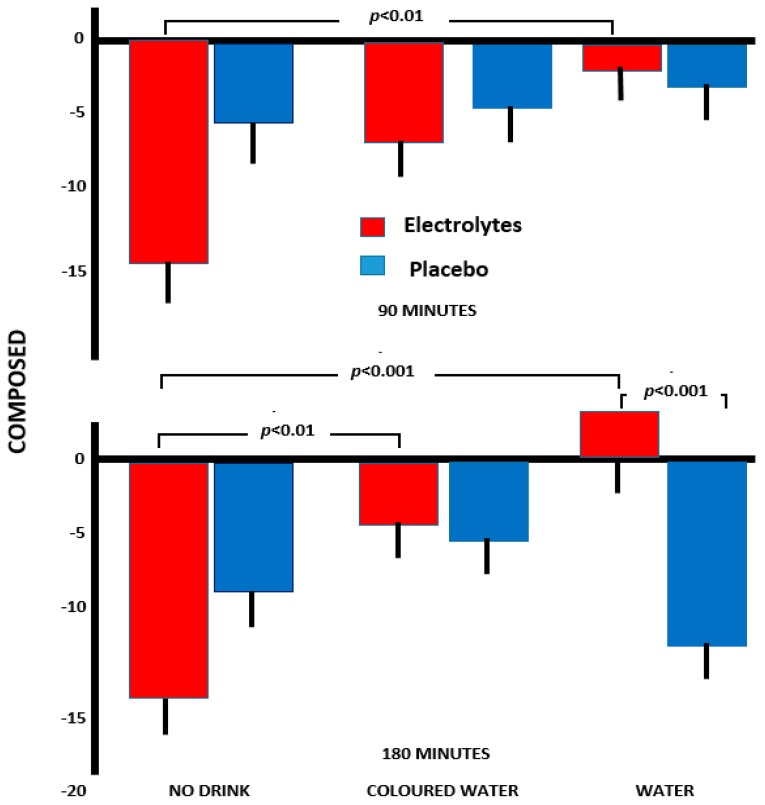
The influence of various drinks on changes over the morning in composure. The data are means +/− standard errors. A minus score reflects increased anxiety.

With ratings of being composed rather than anxious, the interaction Drink×Capsule×Time (*F*(2, 157) = 3.82, *p* < 0.02) reached significance. At 90 min, those who received electrolyte alone reported a significantly greater decline in composure compared to those who received electrolyte combined with plain water (*p* < 0.01, Figure 2). However, after 180 min, consuming electrolyte with water resulted in feeling more composed than after either electrolyte alone (*p* < 0.001) or plain water alone (*p* < 0.001). In addition, those who received coloured water plus electrolyte reported significantly less of a decline in ratings of composure than those who received electrolyte but no drink (*p* < 0.01, Figure 2).

Again, the combination of electrolyte and water most effectively prevented an increase in anxiety. By 180 min, those who received electrolyte plus water reported an overall increase in ratings of composure, something not observed with coloured water.

#### 3.2.3. Clearheaded

With ratings of being clearheaded as opposed to confused, a significant two-way interaction, Time×Drink, resulted (*F*(2,155) = 3.44, *p* < 0.03). Those who received no drink experienced a significant decline in clearheadedness between 90 and 180 min (*p* < 0.03), whereas those who received water (plain or coloured) did not produce a statistically significant decline.

#### 3.2.4. Energy

With rating of being energetic rather than fatigued, a significant interaction, Capsule×Drink (*F*(2,156) = 6.38, *p* < 0.002), was found. Those who had neither a drink nor electrolyte had a greater decline in energy than those who had the electrolyte capsule but no drink (*p* < 0.01). Those who had coloured drink and electrolyte had a greater decline in energy than those who had a coloured drink and no electrolyte (*p* < 0.03).

**Figure 3:**
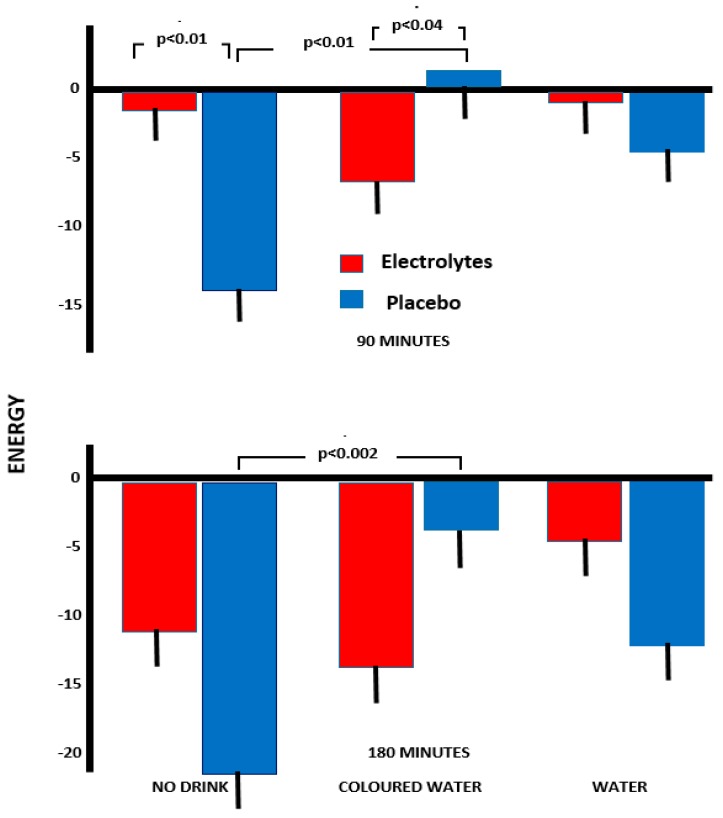
The influence of various drinks on changes over the morning in energy. The data are means +/− standard errors with a minus figure indicating decreased energy.

At 90 min, having received coloured water but no electrolyte was associated with the highest ratings of subjective energy; levels were significantly higher than with coloured water and electrolyte (*p* < 0.04) and those who received no water with no electrolyte (*p* < 0.01, Figure 3).

By 180 min, those who received coloured water plus no electrolyte reported the highest ratings of subjective energy. Participants who received coloured water and the placebo capsule reported significantly less of a decline in energy compared to those who received no drink plus placebo (*p* < 0.002, Figure 3). In a similar manner to previous moods, there was a trend for those who received electrolyte plus water to report less of a decline in energy than those who received electrolyte alone, or electrolyte plus coloured water (Figure 3).

#### 3.2.5. Elation

No significant effects were found with either Drink or Capsule when reports of feeling elated rather than depressed were examined.

#### 3.2.6. Confidence

With ratings of being confident rather than unsure, no significant effects included Drink or Capsule.

### 3.3. The Effect of the Interventions upon Cognition

#### 3.3.1. Arrow Flankers—Congruent Trials (<<<<<)

Analysis of variance was calculated using response times for the arrow flankers test as the dependent variable: Drink (no water, water, coloured water)×Capsule (electrolyte/placebo)×Time (change after 90 and 180 min). A significant effect of capsule was found (*F*(1,142) = 4.07, *p* < 0.05), where those who received electrolyte rather than a placebo had faster response times. In addition, at both 90 (*p* < 0.02) and 180 min (*p* < 0.03), participants who received electrolyte plus water had significantly faster response times than those drinking coloured water plus electrolyte.

**Figure 4:**
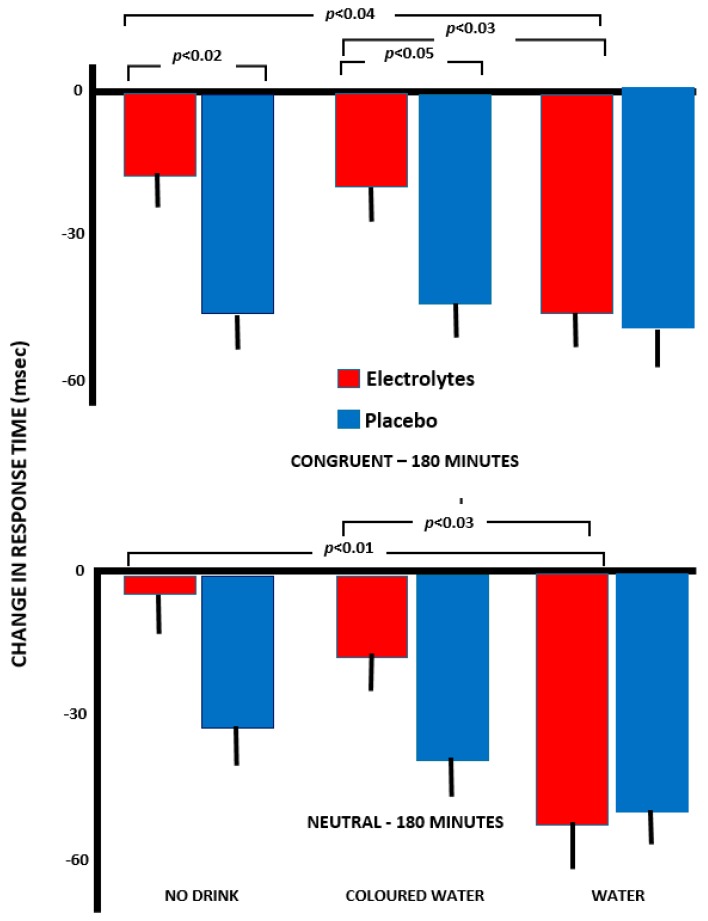
Changes in response times for neutral and congruent trials of the arrow flankers task. The scores are in milliseconds and are changes from baseline values. A lower negative number indicates faster response times. The data are means +/− standard error.

At 180 min, those who received electrolyte plus no water were significantly slower than those who received no water and placebo (*p* < 0.02; Figure 4). Participants who received coloured water plus electrolyte were significantly slower than those who received coloured water alone (*p* < 0.05; Figure 4). The electrolyte has a determental effect except when combined with plain water.

#### 3.3.2. Incongruent Trials (<<><<)

With the incongruent trials, there was a significant effect of the Type of Drink (*F*(2, 141) = 4.17, *p* < 0.02). While response time scores for all groups became quicker between 90 and 180 min, only for those who received plain water did this effect achieve statistical significance (*p* < 0.007).

#### 3.3.3. Neutral Trials (□□<□□)

With neutral trials, a significant Time×Capsule interaction was found *F*(1,141) = 6.26, *p* < 0.01). When no electrolyte was consumed, at 180 min, the response times were significantly slower than at 90 min (*p* < 0.001). At 180 min, response times were significantly quicker when electrolyte had been consumed (*p* < 0.05).

At 90 min, those who received electrolyte plus water had significantly faster response times than those who received electrolyte alone (*p* = 0.003) or electrolyte plus coloured water (*p* < 0.02). Similarly, at 180 min (Figure 4), those who received electrolyte plus water were faster than those who received only electrolyte (*p* < 0.01) or electrolyte plus coloured water (*p* < 0.03) (Figure 4).

#### 3.3.4. Memory

When episodic memory was monitored, change scores were calculated between baseline and 90 min and baseline and 180 min using the sum of recalled words. On no occasion did the nature of the drink or the capsule influence memory.

#### 3.3.5. Serial Sevens

To explore the effect of the intervention upon working memory, a three-way analysis of variance (Drink×Capsule×Time) was conducted using response times. Change scores were calculated between baseline and 90 min, and baseline and 180 min. A significant three-way interaction of Time×Capsule×Drink resulted (*F*(1,154) = 3.10, *p* < 0.05). At 90 min, those who received coloured water were significantly faster than those who received only a placebo (*p* < 0.01). However, after 180 min, those who had received plain water plus electrolyte had the fastest response times which were significantly faster than at 90 min (*p* < 0.001). In contrast, the responses of those who received no electrolyte and no drink were quicker at 180 compared to 90 min (*p* < 0.003). Similarly, the responses of those with coloured water and no electrolyte also slowed (*p* < 0.02). Over the morning, a combination of water and electrolyte was associated with the greatest improvement in response times.

#### 3.3.6. Reaction Times

With the reaction time task, with both one or two lamps, the interventions produced no significant effects, however, the more demanding four and eight lamp variants were influenced. With four lamps, there was a significant Time×Capsule interaction (*F*(1, 160) = 4.10, *p* < 0.05). The reaction times of those who received electrolyte became quicker between 90 and 180 min. In contrast, the reaction times of those who did not receive electrolyte slowed.

With 8 lamps, a Drink×Capsule×Time interaction resulted (*F*(2,153) = 5.46, *p* < 0.005). At 90 min, those that received coloured water plus electrolyte were significantly faster than those that received neither a drink nor electrolyte (*p* < 0.01). There was also a significant difference between 90 and 180 min for those who received no drink plus electrolyte, whereby reaction times were significantly slower (*p* < 0.01).

## 4. Discussion

Supporting previous research, a reduction in body mass of 0.66% was associated with a disruption of functioning (Figure 1, Figure 2 and Figure 3). Previously, in two samples, a loss of 0.55% or 0.59% body mass was associated with increased anxiety [18], and after a loss of 0.72%, drinking plain water improved memory and attention while reducing anxiety [4]. Thus, several findings have shown that a loss body mass of less than 1% was disruptive, although received wisdom has been that a loss of more than 2% was necessary [1,2,3]. The question that arises is whether the consequences of a small reduction in body mass reflect dehydration as such, an adaptation to deal with a minor reduction in body fluid, or alternatively a placebo effect?

The possibility of a cephalic phase in the response to drink has attracted little attention, although presently a different response to plain and red coloured water indicated the influence of psychological mechanisms (Figure 1, Figure 2, and Figure 4). Previously, Johnson and Clydesdale [19] reported that a red colour increased the perceived sweetness of a sugar solution, although this phenomenon may reflect prior experience of the association [20]. It may be important that it was a red colour that was found to be influential (Figure 1, Figure 2, Figure 3 and Figure 4), as red berries and fruits are rich sources of vitamin C, carotenoids and polyphenols, with their antioxidant, anti-inflammatory and anti-viral properties [21,22].

There was evidence of a different response when the water was coloured red (Figure 1, Figure 2, Figure 3 and Figure 4). Although consuming plain water plus electrolyte was associated with greater agreeability and composure (Figure 1 and Figure 2), this did not occur with coloured water. In contrast, drinking the red water was associated with greater energy than when no drink was consumed (Figure 3), something that was not the case with pure water. The clearest findings were with the arrow flankers task (Figure 4) where there were significant differences between drinking pure and coloured water when combined with electrolyte. Although the findings must be viewed as preliminary, the impression is that the expectations associated with a red drink are varied and interact with the task or aspects of mood being considered.

A placebo response, however, was not the entire story. Drinks designed to facilitate the replacement of lost body fluid to a greater extent prevented a decline in mood (Figure 1, Figure 2 and Figure 3), suggesting that both physiological and psychological mechanisms have a role.

Exercise physiology has systematically developed drinks that more efficiently replace lost body fluid. Sodium in a drink helps rehydration, as more fluid is retained and the production of urine declines [23]. Whereas previous studies of hypo-hydration have administered only plain water, for the first time, the present study compared drinks that were predicted to differentially rehydrate. Water alone does not efficiently replace lost fluid as it reduces the sodium concentration in plasma so that the reduced osmolality stimulates diuresis [24]. Therefore, the present study varied the administration of water and sodium chloride while monitoring changes in mood and cognition. A combination of electrolyte and water most successfully prevented an increase in hostility (Figure 1) and anxiety (Figure 2), while attention (Figure 4) and working memory benefitted. The fact that there was a greater impact of a drink that is known to aid rehydration indicated that this is part of the mechanism. In addition, a previous study reported that changes in body mass and osmolality mediated the influence of drinking on cognition [4], offering evidence of the importance of physiological mechanisms. As in this study individuals were unaware of their osmolality, these findings offered additional evidence of the influence of a change in hydration status, irrespective of any placebo effect.

As the present study reduced body mass, it might be natural to assume that dehydration was the underlying mechanism; it is, however, questionable whether a measure of plasma osmolality would have been informative. Although plasma osmolality may be used to demonstrate severe levels of dehydration, it is not sensitive to minor changes in hydration status, as it is closely regulated by homeostatic mechanisms [25,26]. Thus, although plasma osmolality was not assessed, it is unlikely that evidence of tissue dehydration would have resulted. However, although it is unlikely that there are gross changes in tissue dehydration, there must be a mechanism by which a minor loss of body fluid adversely influences mood and cognitive functioning [4,5,6,7,8,9,18] (Figure 1 and Figure 2).

If changes in tissue hydration are not the mechanism, then the possibility arises that homeostatic mechanisms have influences in addition to manipulating fluid levels. A robust finding is that a loss of 0.5% to 0.7% of body mass increases anxiety (Figure 2) [4,18]. A loss of body mass of less than 1%, induced by exercise and a diuretic, increased tension/anxiety [27]. Furthermore, a loss of 0.52% or 0.55% [18] or 0.72% [4] of body mass has also been reported to increase anxiety. In fact, based on brain imaging, a model has been developed whereby inadequate hydration, by selectively modifying the activity of certain areas of the brain, influences autonomic nervous activity with consequences for affect [18].

A French study found that although plasma osmolality was similar in those with a lower rather than higher fluid intake, with a lower intake there were higher levels of plasma cortisol, creatinine and arginine vasopressin [28]. As a loss of plasma volume and the resulting increase in sodium concentration reduces the release of aldosterone, it is relevant that the renin-angiotensin-aldosterone system impacts mood and anxiety [29]. Arginine vasopressin has been suggested to contribute to anxiety and depression [30], and the renin–angiotensin–aldosterone system via angiotensin 1a receptors, has a role in a stress response that involves the hypothalamic–pituitary–adrenal axis [31]. Vian [32] reviewed the evidence associating Angiotensin II with mood. Clinical data, although not as yet double-blind trials, have suggested that drugs, such as anti-hypertensive drugs that block the production of Angiotensin II, help mood disorders and produce a positive outcome in animal models of depression. It has been proposed that aldosterone has a role in depression [33,34,35] and that the level of this hormone predicts the outcome of the disorder [36]. Therefore, future research should monitor the levels of hormones associated with the renin–angiotensin–aldosterone system and relate these to psychological functioning.

A conference of the European Hydration Institute [37] concluded that “regulation is not perfect and dehydration, a positive or negative deviation from the state of euhydration, can occur. Transient mild hypo-hydration is common, and probably of little consequence.” However, at that time, the assumption that transient mild hypo-hydration had few consequences had been subject to little experimentation, although subsequently, evidence has accumulated that minor reductions in hydration have adverse consequences [4,18]. It is not surprising that the adverse effects have been found to be psychological in nature, as it has been argued that the first signs of subclinical nutrient deficiency are typically psychological [38]. As psychological responses are the result of the summated influence of many millions of biochemical processes, even a small reduction in efficiency can have a cumulative effect.

## 5. Conclusions

The potential mechanisms associated with a minor decline in body mass are first a placebo effect, secondly, they result from dehydration as such, and thirdly, they may be secondary consequences of the mechanisms that successfully help the body to respond to a loss of body fluid.

Although plasma osmolality was not assessed, it is unlikely that a measure of plasma osmolality would have demonstrated tissue dehydration, as homeostatic mechanisms have been thought to efficiently regulate small changes in hydration status. However, drinks with or without electrolyte varied in their ability to reverse the impact of a loss of body mass. As the presence of electrolyte influences the ability to rehydrate, it appears that rehydration may have played a role. A possibility to be further considered is that the renin–angiotensin–aldosterone system, while maintaining hydration status, has other influences with psychological consequences.

The fact that, on occasions, there was a different response to plain and coloured water suggests that in addition there were placebo effects. That is prior experience of drinking specific fluids had resulted in psychological expectations and potentially anticipatory physiological responses, although this is a question for future study. Future research should systematically consider the influence of liquids of different colours, aiming to establish their impact on physiological responses that control hydration.

## Figures and Tables

**Table 1:** The influence of six interventions on various indices of hydration status.

Intervention	Baseline Osmolality	Range	Change Osmolality	Range	Percent Change Body Mass	Range
**No Drink**	651.3 (345.5)	94–1115	+101.4 (159.4)	−204–421	−0.67 (0.26)	−1.24–−0.16
**Electrolyte**	*n* = 26		*n* = 21 abcd		*n* = 25 abcd	
**No Drink**	609.9 (257.2)	135–1032	+146.5 (160.1)	−117–508	−0.66 (0.20)	−1.35–−0.42
**Placebo**	*n* = 26		*n* = 25 efgh		*n* = 27 efgh	
**Coloured Water**	640.0 (298.8)	152–1159	−18.7 (193.9)	−314–438	−0.41 (0.30)	−1.28–+0.13
**Electrolyte**	*n* = 30		*n* = 27 ae		*n* = 30 ae	
**Coloured Water**	620.4 (283.1)	128–1058	−20.6 (117.9)	−364–313	−0.41 (0.33)	−1.18–+0.09
**Placebo**	*n* = 29		*n* = 27 bf		*n* = 28 bf	
**Water**	672.1 (308.8)	95–1108	−32.1 (245.3)	−566–405	−0.43 (0.35)	−1.42–+0.02
**Electrolyte**	*n* = 27		*n* = 27 cg		*n* = 27 cg	
**Water**	668.5 (284.5)	131–1163	−0.59 (195.9)	−401–315	0.41 (0.24)	−0.80–+0.15
**Placebo**	*n* = 29		*n* = 27 dh		*n* = 28 dh	

The data are means (standard deviations) with a minus indicating a loss of body mass or reduction in osmolality over the study. The six experimental conditions were compared and where they differed significantly by at least *p* < 0.01, in the vertical columns differences are indicated by pairs of letters. That is, a condition marked ‘a’ differed from another condition marked ‘a’. Osmolality was measured as milli-osmoles per kilogram of solute. The loss of body mass is the percentage change over the duration of the study. Any variation in sample size reflects either technical malfunctioning or the removal of data points as outliers.

## Supplementary Materials

The following are available online at https://www.mdpi.com/2072-6643/11/9/2002/s1, Table S1: The influence of interventions on Agreeableness, Table S2: The influence of interventions on Composure, Table S3: The influence of interventions on Clearheadness, Table S4: The influence of interventions on Elation, Table S5: The influence of interventions on Confidence, Table S6: The influence of interventions on Energy, Table S7: The effect of the interventions upon response times during the arrow flankers task, Table S8: The effect of the intervention upon response times during the serial sevens task, Table S9: The effect of the intervention upon episodic memory, Table S10: The effect of the intervention upon reaction times.
