# Supplementary material for: The Effect of Hypo-Hydration on Mood and Cognition Is Influenced by Electrolyte in a Drink and Its Colour: A Randomised Trial
Source: Nutrients. 2019 Aug 24;11(9):2002. doi: 10.3390/nu11092002 (PMC6769552; doi:10.3390/nu11092002)
Supplement: Supplementary file 1 [file nutrients-11-02002-s001.pdf]

nutrients

SUPPLEMENTARY INFORMATION

An analysis of variance design was used in all cases. 3 X 2 X 2 with the last as a repeated measures (Capsule – Placebo / Electrolyte X Drink – No drink / Plain water / Coloured water) X Time – Baseline / End study).

Table S1. The influence of interventions on Agreeableness

Mood subscale	Effect/Interaction	ANOVA
Agreeableness	Time	*F*(1,154) = 3.042, *p* = 0.083
	Time X Capsule	*F*(1,154) = 1.928, *p* = 0.167
	Time X Drink	*F*(2,154), = 0.417, *p* = 0.660
	Time X Capsule X Drink	*F*(2,154) = 4.510, *p* = 0.012
	Capsule	*F*(1,154) = 1.928, *p* = 0.167
	Drink	*F*(1,154) = 3.599,*p* = 0 .030
	Capsule X Drink	*F*(1,154) = 0.235, *p* = 0.791

Table S2. The influence of interventions on Composure

Mood subscale	Effect/Interaction	ANOVA
Composure	Time	*F*(1,157) = 1.715, *p* =0.192
	Time X Capsule	*F*(1,157) = 6.946, *p* =0.009
	Time X Drink	*F*(2,157) = 1.503, *p* =-.226
	Time X Capsule X Drink	*F*(2,157) = 3.821, *p* =0.024
	Capsule	*F*(1,157) = 0.010, *p* =0.910
	Drink	*F*(2,157) = 3.074, *p* =0.049
	Capsule X Drink	*F*(2,157) = 3.752, *p* =0.026

Table S3. The influence of interventions on Clearheadness

**Mood subscale**	**Effect/Interaction**	**ANOVA**
Clearheadedness	Time	*F*(1,155) = 0 .865, *p* =0.354
	Time X Capsule	*F*(1,155) = 0.193, *p* = 0.661
	Time X Drink	*F*(2,155) = 3.440, *p* = 0.035
	Time X Capsule X Drink	*F*(2,155) = 0.498, *p* = 0.609
	Capsule	*F*(1,155) = 0.467, *p* =0.496
	Drink	*F*(2,155) = 0.322, *p* = 0.718
	Capsule X Drink	*F*(2,155) = 1.333, *p* = 0.267

Table S4. The influence of interventions on Elation

Mood subscale	Effect/Interaction	ANOVA
**Elation**	Time	*F*(1,158) = 11.160, *p*<0 .001
	Time X Capsule	*F*(1,158) = 3.738, *p* =0.055
	Time X Drink	*F*(2,158) = 0.135, *p* =0.873
	Time X Capsule X Drink	*F*(2,158) = 1.410, *p* =0.247
	Capsule	*F*(1,158) = 0.069, *p* =.0793
	Drink	*F*(2,158) = 0.568, *p* =0.558
	Capsule X Drink	*F*(2,158) = 2.243, *p* =0.110

Table S5. The influence of interventions on Confidence

Mood subscale	Effect/Interaction	ANOVA
Confidence	Time	$F(1,153) = 2.360$, p =0.127
	Time X Capsule	$F(1,153) = 0.462$, p =.0.498
	Time X Drink	$F(2,153) = 1.010$, p =0.366
	Time X Capsule X Drink	$F(2,153) = 0.701$, p =0.495
	Capsule	$F(1,153) = 0.600$, p =0.440
	Drink	$F(2,153) = 4.681$, p =0.011
	Capsule X Drink	$F(2,153) = 1.314$, p =0.272

Table S.6 The influence of interventions on Energy

Mood subscale	Effect/Interaction	ANOVA
Energy	Time	$F(1,156) = 18.460$, p <0.001
	Time X Capsule	$F(1,156) = 0.002$, p =0.964
	Time X Drink	$F(1,156) = 0.703$, p =0.497
	Time X Capsule X Drink	$F(1,156) = 0.228$, p =0.796
	Capsule	$F(1,156) = 1.417$, p = 0.263
	Drink	$F(1,156) = 2.545$, p = 0.082,
	Capsule X Drink	$F(1,156) = 6.384$, p =0.002 .

Table S.7 THE EFFECT OF THE INTERVENTIONS UPON RESPONSE TIMES DURING THE ARROW FLANKERS TASK

Cognitive task	Effect/Interaction	ANOVA
**Congruent**	Time	$F(1, 141) = 10.764$, p =0 .001
	Time X Drink	$F(2,142) = 1.579$, $p = 0.223$
	Time X Capsule	$F(1,142) = 2.903$, $p = 0.089$
	Time X Drink X Capsule	$F(2,142) = 0.007$, $p = 0.993$
	Time	$F(2, 141) = 4.165$, $p = 0.017$
	Capsule	$F(2,142) = 2.618$, $p = 0.072$
	Drink X Capsule	$F(2,142) = 1.437$, $p = 0.241$

Cognitive task	Effect/Interaction	ANOVA
**Incongruent**	Time	$F(1, 141) = 10.764$, $p = 0.001$
	Time X Drink	$F(2,141) = 0.339$, $p = 0.713$
	Time X Capsule	$F(1,141) = 2.293$, $p = 0.132$
	Time X Drink X Capsule	$F(2,141) = 0.114$, $p = 0.132$
	Drink	$F(2, 141) = 4.165$, $p = 0.017$
	Capsule	$F(1,141) = 1.106$, $p = 0.295$,
	Drink X Capsule	$F(2,141) = 0.071$, $p = 0.931$

.

Cognitive task	Effect/Interaction	ANOVA
**Neutral Trials**	Time	(F(1,141) = 13.877, p <.001
	Time X Drink	(F(2,141) = 0.831, p = 0.438
	Time X Capsule	F(1,141) = 6.26, p = 0.013
	Time X Drink X Capsule	(F(2,141) = 0.074, p = 0.929
	Drink	(F(2,141) = 5.606, p = 0.005
	Capsule	(F(1,141) = 1.576, p = 0.211
	Drink X Capsule	(F(2,141) = 1.670, p = 0.192

.

Table S8. THE EFFECT OF THE INTERVENTION UPON RESPONSE TIMES DURING THE SERIAL SEVENS TASK

Cognitive task	Effect/Interaction	ANOVA
**Serial sevens**	Time	F(1,154) = 43.030, p <.001
	Time X Drink	F(2,154) = 1.253, p = 0.289
	Time X Capsule	F(2,154) = 0.362, p = 0.548
	Time X Drink X Capsule	F(2,154) = 4.303, p = 0.046
	Drink	F(2,154) = 1.538, p = 0.218
	Capsule	F(1,154) = 0.025, p = 0.874
	Drink X Capsule	F(2,154) = 1.841, p = 0.162

Table S.9 THE EFFECT OF THE INTERVENTION UPON EPISODIC MEMORY

Mood subscale	Effect/Interaction	ANOVA
	Time	$F(1,165)=8.915$, $p = 0.003$
	Time X Drink	$F(2,165) =2.262$, $p =0$ .107
	Time X Capsule	$F(1,165) = 0.208$, $p= 0.649$
	Time X Capsule X Drink	$F(2,165)= 0.877$, $p =0.417$
	Memory type	$F(1,165) = 90.995$, p .001
	Memory type X Drink	$F(2,165) = 0.617$, $p=0.541$
	Memory type X Capsule	$F(1,165) = 1.747$, $p=0.188$
	Memory type X Capsule X Drink	$F(2,165)=0.876$, $p =0.418$
	Time X Memory type	$F(1,165)=12.082$, $p =.001$
	Time X Memory type X Drink type	$F(2,165) = 1.014$, $p=.365$
	Time X Memory type X Capsule	$F(1,165) = 0.647$, $p =.413$
	Time X Memory type X Drink type X Capsule	$F(2,165) = 0.046$, $p =.955$
	Capsule	$F(1,165) = 0.454$, $p = .636$
	Drink	$F(1,165) =0$ .009, $p = .923$
	Capsule X Drink	$F(1,165) = 0.800$, $p = .451$

Note. Type of memory is whether recall was immediately or after 20 minutes

Table S.10 THE EFFECT OF THE INTERVENTION UPON REACTION TIMES

Lamp	Effect/Interaction	ANOVA
1	Time	(F(1, 160) = 0.542, p = 0.463)
1	Time X Drink	(F(2, 160) = 0.118, p = 0.889)
1	Time X Capsule	(F(1, 160) = 1.197, p = 0.275)
1	Time X Capsule X Drink	(F(2, 160) = 0.098, p = 0.907)
2	Time	(F(1, 160) = 1.393, p = 0.240)
2	Time X Drink	(F(2, 160) = 0.601, p = 0.549)
2	Time X Capsule	(F(1, 160) = 0.724, p = 0.384)
2	Time X Capsule X Drink	(F(2, 160) = 0.328, p = 0.721)
4	Time	(F(1, 160) = 0.498, p = 0.482)
4	Time X Drink	(F(2, 160) = 1.104, p = 0.334)
4	Time X Capsule	(F(1, 160) = 4.097, p = 0.045)
4	Time X Capsule X Drink	(F(2, 160) = 0.409, p = 0.605)
8	Time	(F(1, 153) = 0.013, p = 0.653)
8	Time X Drink	(F(2, 153) = 1.203, p = 0.398)
8	Time X Capsule	(F(1, 153) = 0.483, p = 0.962)
8	Time X Capsule X Drink	(F(2, 153) = 3.938, p = 0.005)

CONSORT
TRANSPARENT REPORTING of TRIALS

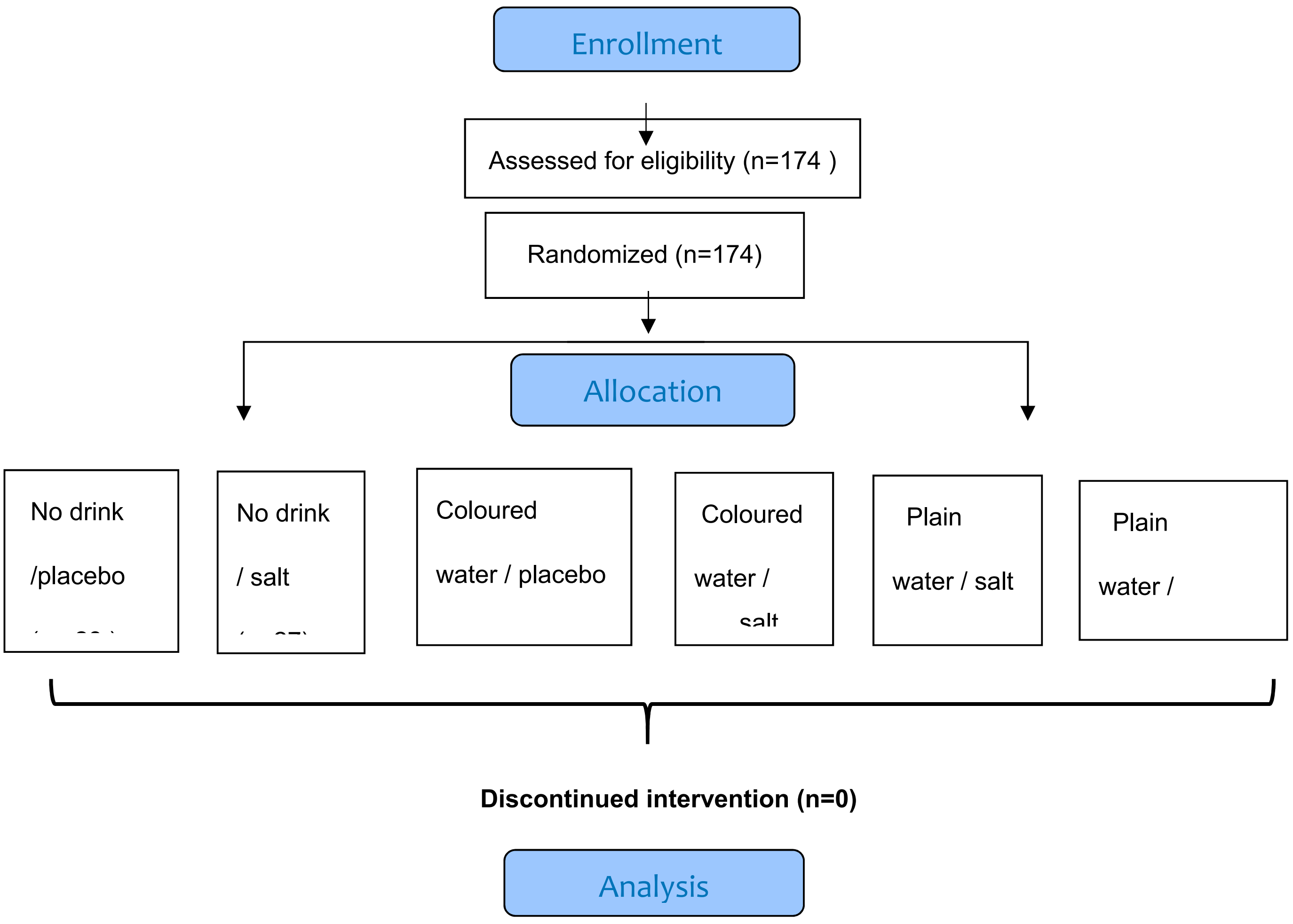

Outliers

Agreeable n=0 n=4 n=2 n=0 n=1 n=2

Composed n=1 n=0 n= 1 n=1 n=1 n= 3

Clearheaded n=2 n=0 n=0 n=3 n=1 n= 2

Energetic n= 0 n=1 n=2 n=2 n=0 n=2

Elated n= 1 n= 0 n= 1 n=1 n=2 n= 3 Confident n=3 n= 2 n=0 n=2 n=1 n=2
